# The predictive value of NT-proBNP/ALB ratio for in-hospital pericardial effusion in young acute myocardial infarction patients

**DOI:** 10.3389/fcvm.2026.1828408

**Published:** 2026-06-24

**Authors:** Hang Yu, Shanshan Qi, Gang Tian

**Affiliations:** 1 Department of Cardiology, The First Affiliated Hospital of Xi’an Jiaotong University, Xi’an, China; 2 Department of Health Sciences, The First Affiliated Hospital of Xi’an Jiaotong University, Xi’an, China

**Keywords:** acute myocardial infarction, NT-proBNP/albumin ratio, pericardial effusion, plateletcrit, young adults

## Abstract

**Background:**

The incidence of acute myocardial infarction (AMI) in young adults (<45 years) is increasing. Pericardial effusion (PE) is a notable complication reflecting hemodynamic instability and severe inflammation; however, sensitive biomarkers for early risk stratification in this specific demographic remain under-investigated. The N-terminal pro-B-type natriuretic peptide to albumin ratio (NT-proBNP/ALB) integrates hemodynamic load with inflammatory-nutritional status, yet its prognostic utility for PE in young AMI patients is unexplored.

**Methods:**

This retrospective observational study enrolled 1,974 young AMI patients admitted to the First Affiliated Hospital of Xi'an Jiaotong University between January 2018 and October 2025. Patients were categorized into PE and non-PE groups based on echocardiographic findings. Multivariate logistic regression was utilized to identify independent predictors. The incremental predictive value of the log-transformed NT-proBNP/ALB ratio (lg BNP/ALB) and Plateletcrit (PCT, a platelet mass marker) was evaluated using Receiver Operating Characteristic (ROC) curves, Net Reclassification Improvement (NRI), and Integrated Discrimination Improvement (IDI).

**Results:**

In-hospital PE occurred in 7.1% (141/1,974) of the cohort. Multivariate analysis identified lg (NT-proBNP/ALB) (adjusted OR: 3.23, 95% CI: 1.30–5.78, *P* < 0.001) and PCT (adjusted OR: 1.87, 95% CI: 1.08–3.23, *P* = 0.025) as independent risk factors, alongside STEMI, LVEF, eGFR, and total cholesterol. The incorporation of lg (NT-proBNP/ALB) and PCT into a baseline clinical model significantly improved the Area Under the Curve (AUC) from 0.718 to 0.849 (*P* < 0.001). Furthermore, reclassification analyses confirmed significant improvements in risk stratification, with a categorical NRI of 0.2198 (*P* < 0.001) and an IDI of 0.1116 (*P* < 0.001).

**Conclusion:**

The admission NT-proBNP/ALB ratio and PCT were independently associated with in-hospital pericardial effusion in young AMI patients and provided incremental value for early risk stratification beyond conventional clinical indicators.

## 1 Introduction

Acute myocardial infarction (AMI) remains a leading cause of cardiovascular mortality and morbidity worldwide. Traditionally regarded as a disease predominantly affecting the elderly, AMI in young adults (typically defined as <45 years) has shown a worrying upward trend over the past decades, driven by the growing prevalence of unhealthy lifestyles (e.g., smoking, excessive alcohol consumption, sedentary behavior) and metabolic risk factors (e.g., obesity, hypertension, type 2 diabetes mellitus) (1, 2). Unlike elderly AMI patients, young patients exhibit distinct pathophysiological characteristics, including fewer coronary collateral vessels, a more intense systemic inflammatory response, and unique atherosclerotic plaque morphology (e.g., a higher prevalence of plaque erosion) (3). Although young AMI patients generally have a better short-term survival rate, in-hospital complications can trigger adverse left ventricular remodeling and poor long-term prognosis, highlighting the necessity of accurate and early risk stratification for this population (4).

Pericardial effusion (PE) is a notable and severe complication following AMI, often indicative of transmural myocardial necrosis, severe post-infarction inflammation, or underlying hemodynamic instability (5). Clinically, effusions are graded as small (<1 cm, corresponding to <100 mL), medium (1–2 cm, corresponding to 100–500 mL), large (>2 cm, corresponding to >500 mL), and very large (>2.5 cm, corresponding to >700 mL) as determined by the largest size of the end-diastolic echo-free space surrounding the heart (6). Small PE is usually a self-limiting condition, but medium to very large PE is closely associated with an increased risk of life-threatening adverse events, including left ventricular free wall rupture, cardiac tamponade, and higher in-hospital mortality (7). Young AMI patients are prone to developing an intense “inflammatory storm” in the acute phase of infarction, which may lead to distinct mechanisms of pericardial fluid exudation compared with the elderly (8) Therefore, identifying sensitive and specific biomarkers to predict the onset of PE in young AMI patients is crucial for timely clinical intervention and close monitoring.

N-terminal pro-B-type natriuretic peptide (NT-proBNP) is a well-recognized gold standard biomarker for evaluating ventricular wall stress and cardiac hemodynamic dysfunction. Elevated NT-proBNP levels in the acute phase of AMI are strongly correlated with infarct size and left ventricular filling pressure, directly reflecting the degree of cardiac structural and functional damage (9–11). In contrast, serum albumin (ALB), beyond its role as a classic nutritional marker, is an important negative acute-phase protein and a key determinant of plasma colloid osmotic pressure. Hypoalbuminemia in the acute phase of AMI not only reflects the severity of systemic inflammation but also reduces plasma colloid osmotic pressure, thereby promoting fluid leakage from the vascular space to extravascular spaces, including the pericardial cavity (12). Although both NT-proBNP and ALB have independent prognostic value in acute coronary syndromes, single biomarkers are often susceptible to various confounding factors (e.g., renal function, nutritional status), leading to limited predictive accuracy.

The NT-proBNP/ALB ratio has recently emerged as a novel composite biomarker that integrates hemodynamic load, inflammatory status, and nutritional reserves into a single parameter. In various cardiovascular diseases such as chronic heart failure and septic cardiomyopathy, this ratio has been proven to have superior predictive value for adverse events compared with single NT-proBNP or ALB (13). However, the prognostic utility of the NT-proBNP/ALB ratio for the development of in-hospital PE in young AMI patients has not been explored. Considering the unique interaction between high inflammatory burden and severe hemodynamic stress in this population, we hypothesized that the NT-proBNP/ALB ratio could serve as a sensitive and specific predictor for in-hospital PE in young AMI patients.

Therefore, the present retrospective study aimed to evaluate the association between the admission NT-proBNP/ALB ratio and the occurrence of in-hospital PE in AMI patients aged <45 years. By determining the predictive threshold and clinical efficacy of this simple, cost-effective composite biomarker, we intended to provide a practical tool for clinicians to identify young AMI patients at high risk of PE and optimize the early risk stratification and clinical management strategies for this specific population.

## 2 Materials and methods

### 2.1 Study population

This retrospective observational study was conducted at the First Affiliated Hospital of Xi'an Jiaotong University. We screened all patients admitted to the Coronary Care Unit (CCU) or Department of Cardiology with a confirmed diagnosis of AMI from January 2018 to October 2025. We consecutively enrolled all patients meeting the inclusion criteria during the study period to minimize selection bias.

#### 2.1.1 Inclusion criteria

(1) Confirmed diagnosis of AMI in accordance with the Fourth Universal Definition of Myocardial Infarction; (2) Age < 45 years at admission; (3) Completion of transthoracic echocardiography (TTE) within 48–72 h of admission to assess pericardial status; additional TTE examinations during hospitalization were performed when clinically indicated according to routine practice. (4) Availability of essential clinical, laboratory, and echocardiographic data, with limited missingness handled by multiple imputation.

#### 2.1.2 Exclusion criteria

(1) Pre-existing pericardial effusion, pericarditis, or a history of pericardial surgery before AMI onset; (2) AMI caused by non-ischemic etiologies (e.g., chest trauma, major surgery, aortic dissection); (3) Severe comorbidities that could independently affect pericardial fluid accumulation (e.g., malignant tumors, active tuberculosis, severe autoimmune diseases, end-stage renal disease); (4) Incomplete medical records or missing key echocardiographic parameters and laboratory indicators.

**Figure 1:**
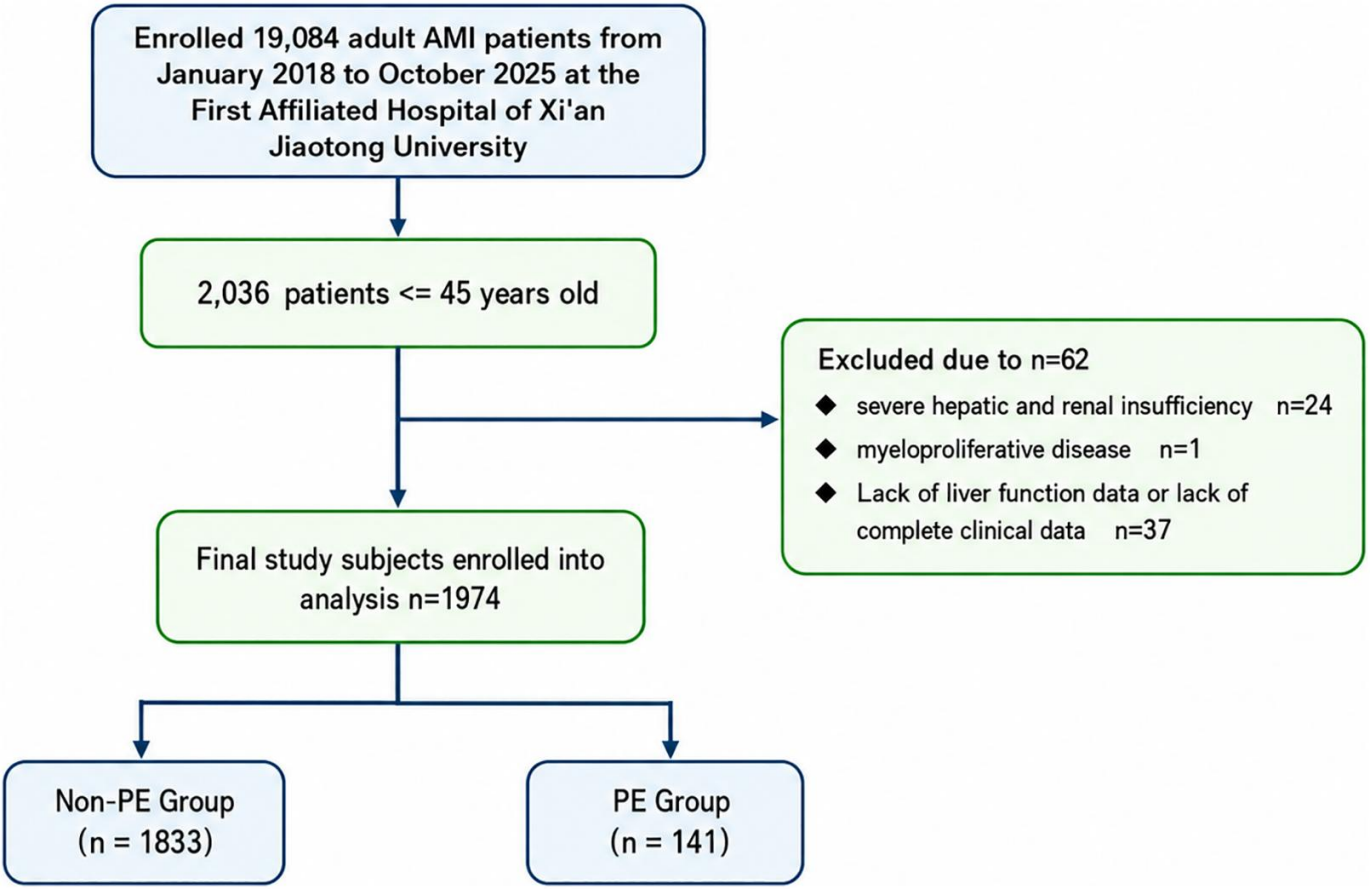
Flowchart of patient enrollment and study population selection. From January 2018 to October 2025, 19084 adult AMI patients were enrolled from the First Affiliated Hospital of Xi'an Jiaotong University. Among them, 2036 patients were ≤ 45 years old. After excluding 62 patients due to severe hepatic and renal insufficiency (n = 24), myeloproliferative disease (n = 1), and lack of liver function data or complete clinical data (n = 37), 1974 patients were finally included in the study analysis. These patients were divided into a Non-PE group (n = 1833) and a PE group (n = 141).

Eligible patients were categorized into the PE group (new-onset in-hospital PE) and the non-PE group according to TTE findings obtained during hospitalization, including the initial examination and any clinically indicated follow-up assessments when available (Figure 1).

### 2.2 Ethics statement

The study protocol was reviewed and approved by the Ethics Committee of the First Affiliated Hospital of Xi'an Jiaotong University (Approval No. XJTU1AF2025LSYY-728). Due to the retrospective nature of the study, which only involved the analysis of existing, de-identified clinical data, the requirement for written individual informed consent was waived by the institutional review board.

### 2.3 Data collection

All relevant clinical data were extracted from the hospital's unified electronic medical record (EMR) system, including: demographic information [age, gender, body mass index (BMI)]; clinical characteristics [Killip class, comorbidities, infarction location, ST-segment elevation myocardial infarction (STEMI)/non-STEMI, heart rate, blood pressure]; laboratory parameters (routine blood tests, biochemical markers, coagulation profiles, NT-proBNP, ALB, platelet indices); and echocardiographic data [left ventricular ejection fraction (LVEF), left ventricular end-diastolic diameter (LVEDD), pericardial status].

All laboratory samples (NT-proBNP, ALB, PCT, etc.) were collected from fasting venous blood on the morning after admission, with all detections completed in the hospital's central clinical laboratory using standardized methods. The lg (NT-proBNP/ALB) ratio was calculated as the base-10 logarithm of the ratio of NT-proBNP (pg/mL) to ALB (g/L) levels.

### 2.4 Definitions and outcome measures

#### 2.4.1 Acute myocardial infarction (AMI)

Defined according to the Fourth Universal Definition of Myocardial Infarction, characterized by a rise and/or fall of cardiac troponin (cTn) values with at least one value above the 99th percentile upper reference limit (URL), combined with clinical evidence of myocardial ischemia [typical ischemic chest pain, characteristic electrocardiogram (ECG) changes, or imaging evidence of new loss of viable myocardium].

#### 2.4.2 Primary outcome

Development of new-onset in-hospital PE after AMI onset. PE was diagnosed by experienced sonographers using TTE, defined as the presence of a persistent echo-free space between the visceral and parietal pericardium throughout the cardiac cycle. All sonographers responsible for TTE diagnosis and PE quantification were blinded to the patients' clinical and laboratory data to avoid detection bias.

### 2.5 Statistical analysis

Continuous variables were first assessed for normality using the Kolmogorov–Smirnov test. Variables conforming to a normal distribution were expressed as mean ± standard deviation (SD) and compared between groups using the *t*-test. Non-normally distributed variables were presented as median with interquartile range (IQR) (25th–75th percentile) and compared using the Mann–Whitney U test. Categorical variables were expressed as frequencies and percentages [*n* (%)] and compared using the Chi-square (*χ*²) test or Fisher's exact test, as appropriate. Prior to regression analyses, the proportion of missing data was evaluated. Missing values, which accounted for <5% of the total dataset, were handled using multiple imputation.

To identify risk factors associated with in-hospital pericardial effusion (PE), univariate logistic regression analysis was initially performed on candidate variables (including demographic, clinical, and laboratory factors). Variables with *P* < 0.05 in univariate analysis and clinically relevant covariates were considered for inclusion. Two multivariable models were then constructed: a baseline clinical model (Model 1) and an extended biomarker-enriched model (Model 2). The results are presented as odds ratios (ORs) with their corresponding 95% confidence intervals (CIs). The discriminative performance of the predictive models was evaluated using Receiver Operating Characteristic (ROC) curves, and the Area Under the Curve (AUC) was calculated. The DeLong test was employed to compare the AUCs between different models. To further assess the incremental predictive value of the novel biomarkers (lg BNP/ALB ratio and PCT), the Net Reclassification Improvement (NRI) and Integrated Discrimination Improvement (IDI) were calculated. To reduce the risk of model overfitting, the number of candidate predictors included in the multivariable model was restricted based on clinical relevance and statistical significance in univariate analysis, ensuring an acceptable events-per-variable ratio. Sensitivity considerations regarding model stability were also taken into account when constructing the final model. As a sensitivity analysis, LASSO logistic regression with 10-fold cross-validation was performed to further assess model stability and the robustness of variable selection. Data analyses were performed using SPSS software (version 26.0, IBM Corp., Armonk, NY, USA) and R software (version 4.4.2, R Foundation for Statistical Computing). Figures were generated using GraphPad Prism 9.0 (GraphPad Software Inc., San Diego, CA, USA). A two-sided *P*-value < 0.05 was considered statistically significant.

## 3 Results

### 3.1 Baseline characteristics of the study population

A total of 1,974 young patients (<45 years) with acute myocardial infarction were included, of whom 141 (7.1%) developed in-hospital pericardial effusion (PE). Baseline characteristics are summarized in Table 1.

**Table 1:** Baseline characteristics and comorbidities of study patients with AMI.

Variables	Total (*N* = 1,974)	Non-PE Group (*n* = 1,833)	PE Group (*n* = 141)	*P* Value
Demographics				
Age, years (Mean ± SD)	38.96 ± 4.96	39.00 ± 4.90	38.36 ± 5.61	0.139
Male, *n* (%)	1,873 (94.9%)	1,746 (95.3%)	127 (90.1%)	0.032
BMI, kg/m²	26.98 ± 4.03	27.11 ± 4.03	25.27 ± 3.57	<0.001*
Risk Factors				
Hypertension, *n* (%)	1,807 (91.5%)	1,673 (91.2%)	134 (95.0%)	0.012
Diabetes mellitus, *n* (%)	459 (23.3%)	415 (22.6%)	44 (31.2%)	0.020
Arrhythmia, *n* (%)	153 (7.8%)	135 (7.4%)	18 (12.8%)	0.021
Current Smoking, *n* (%)	1,587 (80.4%)	1,483 (80.8%)	104 (73.8%)	0.039
Clinical Characteristics				
Anterior wall MI, *n* (%)	573 (29.0%)	500 (27.3%)	73 (51.8%)	<0.001*
Killip Class ≥ II, *n* (%)	453 (22.9%)	390 (21.3%)	63 (44.7%)	<0.001*
STEMI, *n* (%)	1,214 (61.5%)	1,112 (60.7%)	102 (72.3%)	0.006
Received thrombolysis, *n* (%)	218 (11.0%)	203 (11.1%)	15 (10.6%)	0.873
SBP, mmHg	133.56 ± 25.39	135.02 ± 24.56	129.25 ± 27.59	0.037
DBP, mmHg	88.10 ± 17.97	88.45 ± 17.27	87.08 ± 19.44	0.480
Heart rate, bpm	82.69 ± 15.51	82.03 ± 15.06	91.39 ± 18.68	<0.001*
NT-proBNP, pg/mL	377.15 (132.60, 864.00)	346.49 (121.60, 787.85)	1,591.00 (605.90, 3,195.00)	<0.001*
hs-CRP, mg/L	4.40 (1.62, 7.38)	4.15 (1.59, 7.15)	6.00 (2.88, 10.00)	<0.001*
AST, U/L	46.50 (26.00, 101.00)	46.00 (26.00, 98.00)	55.00 (26.50, 156.00)	0.051
ALT, U/L	41.00 (27.00, 60.00)	40.00 (26.00, 58.00)	51.00 (31.50, 77.00)	<0.001*
TBIL, μmol/L	15.40 (11.40, 21.50)	15.40 (11.40, 21.36)	16.00 (10.90, 22.80)	0.723
ALB, g/L	40.76 ± 5.08	41.00 ± 4.88	37.64 ± 6.46	<0.001*
RBC, 10^12^/L	5.29 ± 1.42	5.35 ± 1.07	5.33 ± 2.16	0.591
RDW, %	43.10 ± 3.71	42.97 ± 3.58	44.78 ± 4.78	<0.001*
WBC, 10^9^/L	10.02 ± 3.52	9.97 ± 3.40	10.71 ± 4.70	0.017
NEU, 10^9^/L	7.41 ± 3.30	7.36 ± 3.22	8.10 ± 4.16	0.010
PLT, 10^9^/L	238.71 ± 69.45	237.71 ± 66.80	251.79 ± 97.10	0.022
CK, U/L	253.00 (95.00, 817.25)	252.00 (96.00, 801.00)	296.00 (80.50, 1,030.00)	0.758
CKMB, U/L	26.00 (14.70, 77.25)	26.00 (14.93, 76.85)	24.50 (12.70, 85.50)	0.487
CTNT, ng/mL	0.41 (0.06, 1.48)	0.38 (0.06, 1.40)	0.99 (0.18, 2.99)	<0.001*
APTT, s	31.95 ± 7.94	31.95 ± 7.94	32.00 ± 7.42	0.953
PTA, %	100.17 ± 18.19	100.72 ± 17.91	93.16 ± 20.31	<0.001*
FIB, g/L	3.68 ± 1.47	3.64 ± 1.45	4.16 ± 1.69	<0.001*
INR	1.03 ± 0.21	1.03 ± 0.21	1.10 ± 0.28	<0.001*
BUN, mmol/L	4.90 ± 2.74	4.75 ± 2.21	6.87 ± 2.17	<0.001*
UA, μmol/L	365.62 ± 109.62	365.14 ± 107.73	371.98 ± 132.18	0.482
HCY, umol/L	22.80 (13.20, 39.89)	22.94 (13.30, 39.87)	21.65 (11.84, 41.26)	0.848
EGFR, mL/min/1.73 m^2^	111.02 ± 25.80	111.92 ± 24.43	99.45 ± 37.65	<0.001*
LDLC, mmol/L	2.78 ± 1.02	2.79 ± 1.03	2.62 ± 0.91	0.077
HbA1c, %	6.34 ± 1.64	6.31 ± 1.62	6.73 ± 1.85	0.006
BNP/ALB	9.06 (2.81, 21.95)	8.19 (2.64, 19.85)	41.86 (14.27, 90.77)	<0.001*
LVEF, %	54.8 ± 8.1	55.6 ± 7.6	47.2 ± 6.1	<0.001*
LVEDD, mm	47.9 ± 4.3	47.6 ± 4.1	52.5 ± 3.5	<0.001^*^

Data are presented as mean ± standard deviation, median (interquartile range), or *n* (%).

^*^Indicates statistically significant difference (*P* < 0.05).

HTN, hypertension; HR, heart rate; Avg SBP, average systolic blood pressure; Avg DBP, average diastolic blood pressure; LVEF, left ventricular ejection fraction; LVEDD, left ventricular end-diastolic dimension; STEMI, ST-elevation myocardial infarction; PCI, percutaneous coronary intervention; PTCA, percutaneous transluminal coronary angioplasty; CABG, coronary artery bypass grafting.

No significant difference in age was observed between groups (38.36 ± 5.61 vs. 39.00 ± 4.90 years, *P* = 0.139). The proportion of male patients was lower in the PE group than in the non-PE group [127/141 (90.1%) vs. 1,746/1,833 (95.3%), *P* = 0.032]. In addition, patients who developed PE had a lower body mass index (25.27 ± 3.57 vs. 27.11 ± 4.03 kg/m², *P* < 0.001) and a higher prevalence of hypertension (95.0% vs. 91.2%, *P* = 0.012), diabetes mellitus (31.2% vs. 22.6%, *P* = 0.020), and arrhythmia (12.8% vs. 7.4%, *P* = 0.021), while current smoking was less frequent (73.8% vs. 80.8%, *P* = 0.039).

Clinically, PE patients presented with more severe cardiac impairment, reflected by a higher proportion of Killip class ≥ II (44.7% vs. 21.3%, *P* < 0.001) and a higher incidence of STEMI (72.3% vs. 60.7%, *P* = 0.006). They also exhibited higher heart rates (91.39 ± 18.68 vs. 82.03 ± 15.06 bpm, *P* < 0.001) and slightly lower systolic blood pressure (129.25 ± 27.59 vs. 135.02 ± 24.56 mmHg, *P* = 0.037).

Markers of myocardial injury and systemic inflammation were significantly elevated in the PE group, including NT-proBNP [1,591.00 (605.90, 3,195.00) vs. 346.49 (121.60, 787.85) pg/mL, *P* < 0.001], hs-CRP, CTNT, WBC count, and neutrophil count (all *P* < 0.05).

In addition, patients with PE showed lower albumin levels (37.64 ± 6.46 vs. 41.00 ± 4.88 g/L, *P* < 0.001), reduced renal function (eGFR: 99.45 ± 37.65 vs. 111.92 ± 24.43 mL/min/1.73m², *P* < 0.001), and higher HbA1c levels (6.73 ± 1.85 vs. 6.31 ± 1.62%, *P* = 0.006). Coagulation-related indicators, including fibrinogen and INR, were also significantly increased (both *P* < 0.001).

Notably, the BNP/ALB ratio was markedly elevated in patients who developed in-hospital pericardial effusion [41.86 (14.27, 90.77) vs. 8.19 (2.64, 19.85), *P* < 0.001]. Echocardiographic parameters further showed worse cardiac structure and function in the PE group, with significantly lower LVEF (47.2 ± 6.1% vs. 55.6 ± 7.6%, *P* < 0.001) and larger LVEDD (52.5 ± 3.5 mm vs. 47.6 ± 4.1 mm, *P* < 0.001).

### 3.2 Univariable analysis of risk factors

Univariable logistic regression analysis was performed to identify potential factors associated with the development of in-hospital pericardial effusion (PE) in young patients with acute myocardial infarction. The results are presented in Table 2.

**Table 2:** Univariate logistic regression analysis for in-hospital pericardial effusion.

Variable	OR (95% CI)	*P* Value
Demographics		
Male gender	0.45 (0.25–0.82)	0.009
BMI (kg/m^2^)	0.93 (0.89–0.98)	0.006
T2DM	1.55 (1.07–2.25)	0.021
Clinical Severity		
Heart rate (bpm)	1.04 (1.03–1.05)	<0.001
Killip class ≥ II	2.99 (2.11–4.24)	<0.001
STEMI diagnosis	1.70 (1.16–2.48)	0.007
Anterior wall infarction	2.86 (2.03–4.05)	<0.001
LVEF (per 1% decrease)	1.16 (1.13–1.19)	<0.001
Key Biomarkers		
hs-CRP (mg/L)	1.14 (1.08–1.19)	<0.001
PCT	2.12 (1.39–3.23)	<0.001
eGFR (per 10 units increase)	0.86 (0.81–0.92)	<0.001
TC	1.14 (1.03–1.87)	<0.001
ALB	0.89 (0.86–0.92)	<0.001
lg NT-proBNP	7.08 (5.03–9.98)	<0.001
lg (NT-proBNP/ALB ratio)	6.76 (4.85–9.43)	<0.001

AMI, acute myocardial infarction; BMI, body mass index; CI, confidence interval; eGFR, estimated glomerular filtration rate; hs-CRP, high-sensitivity C-reactive protein; lg, log-transformed; LVEF, left ventricular ejection fraction; NT-proBNP, N-terminal pro-B-type natriuretic peptide; OR, odds ratio; PCT, plateletcrit; STEMI, ST-segment elevation myocardial infarction; TC, total cholesterol; T2DM, Type 2 diabetes mellitus; ALB, albumin.

Among demographic characteristics, male sex (OR: 0.45, 95% CI: 0.25–0.82, *P* = 0.009) and higher BMI (OR: 0.93, 95% CI: 0.89–0.98, *P* = 0.006) were associated with a lower risk of PE, whereas the presence of type 2 diabetes mellitus (OR: 1.55, 95% CI: 1.07–2.25, *P* = 0.021) was associated with an increased risk.

Indicators reflecting clinical severity of myocardial infarction were strongly associated with PE occurrence. Higher heart rate (OR: 1.04, 95% CI: 1.03–1.05, *P* < 0.001), Killip class ≥ II (OR: 2.99, 95% CI: 2.11–4.24, *P* < 0.001), STEMI diagnosis (OR: 1.70, 95% CI: 1.16–2.48, *P* = 0.007), anterior wall infarction (OR: 2.86, 95% CI: 2.03–4.05, *P* < 0.001), and reduced LVEF (per 1% decrease: OR: 1.16, 95% CI: 1.13–1.19, *P* < 0.001) were all significantly associated with increased odds of PE.

Among laboratory parameters, inflammatory and metabolic biomarkers were significantly associated with PE. Higher hs-CRP (OR: 1.14, 95% CI: 1.08–1.19, *P* < 0.001), plateletcrit (PCT) (OR: 2.12, 95% CI: 1.39–3.23, *P* < 0.001), and total cholesterol (OR: 1.14, 95% CI: 1.03–1.87, *P* < 0.001) were associated with increased risk, whereas better renal function (eGFR per 10-unit increase: OR: 0.86, 95% CI: 0.81–0.92, *P* < 0.001) and higher albumin levels (OR: 0.89, 95% CI: 0.86–0.92, *P* < 0.001) were associated with lower risk of PE.

**Figure 2:**
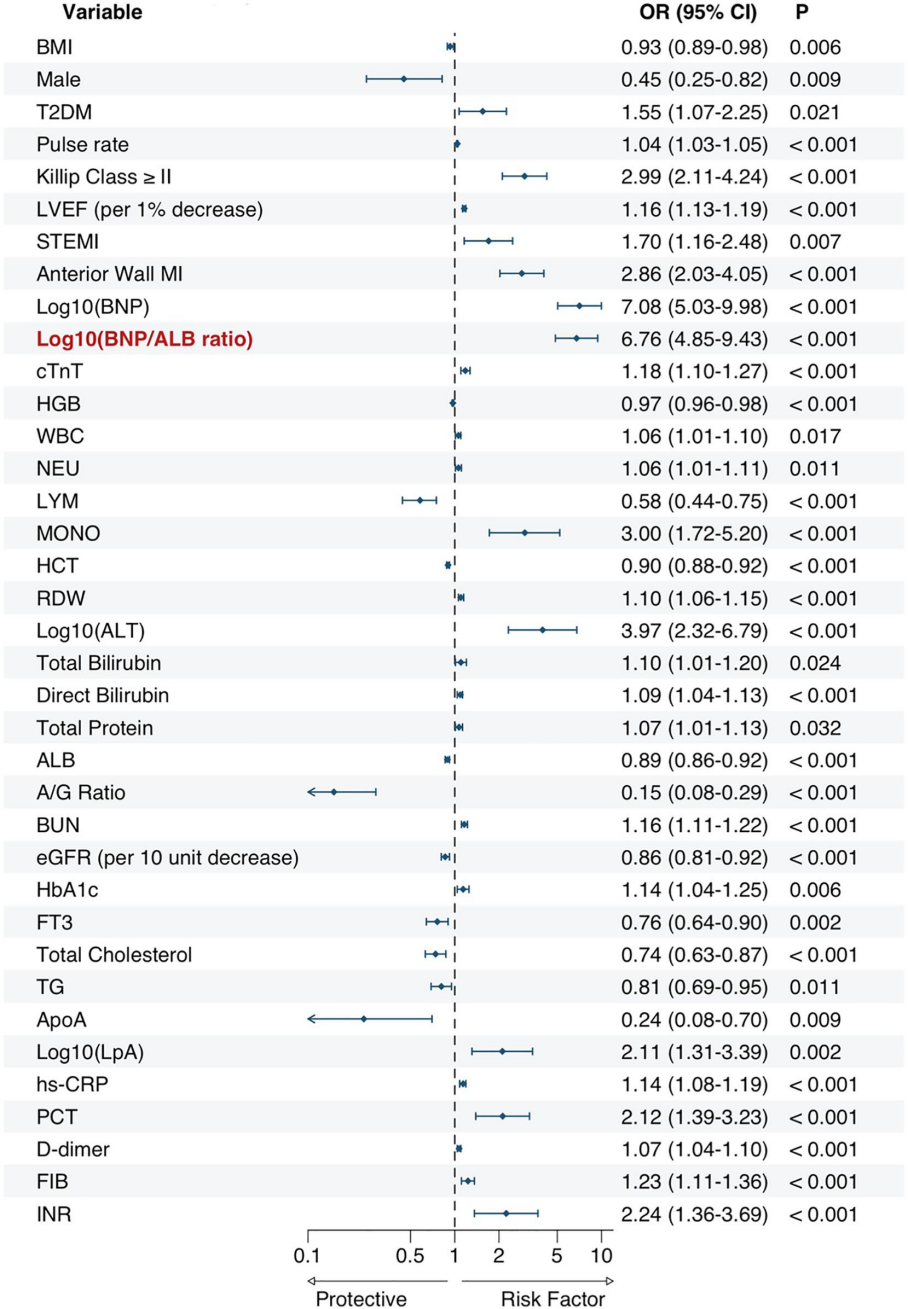
Forest plot of univariate logistic regression analysis for risk factors associated with in-hospital pericardial effusion Note: The forest plot displays the odds ratios (ORs) and 95% confidence intervals (CIs) for selected significant predictors identified in the univariate analysis. Variables are categorized into demographics, clinical characteristics, and laboratory findings. Abbreviations: BMI: body mass index; T2DM: type 2 diabetes mellitus; LVEF: left ventricular ejection fraction; STEMI: ST-segment elevation myocardial infarction; MI: myocardial infarction; BNP: B-type natriuretic peptide; ALB: albumin; cTnT: cardiac troponin T; HGB: hemoglobin; WBC: white blood cell; NEU: neutrophil; LYM: lymphocyte; MONO: monocyte; HCT: hematocrit; RDW: red blood cell distribution width; ALT: alanine aminotransferase; A/G: albumin to globulin; BUN: blood urea nitrogen; eGFR: estimated glomerular filtration rate; HbA1c: glycated hemoglobin; FT3: free triiodothyronine; TG: triglyceride; ApoA: apolipoprotein A; LpA: lipoprotein(a); hs-CRP: high-sensitivity C-reactive protein; PCT: procalcitonin; FIB: fibrinogen; INR: international normalized ratio; OR: odds ratio; CI: confidence interval.

Notably, cardiac biomarker NT-proBNP demonstrated a strong association with PE risk. Log-transformed NT-proBNP showed a markedly elevated odds ratio (OR: 7.08, 95% CI: 5.03–9.98, *P* < 0.001). Consistent with our hypothesis, the log-transformed NT-proBNP/albumin ratio exhibited a robust association with PE (OR: 6.76, 95% CI: 4.85–9.43, *P* < 0.001), suggesting that this composite biomarker may provide incremental value for identifying patients at increased risk of pericardial effusion (Figure 2).

### 3.3 Multivariable analysis of risk factors

Multivariable logistic regression analysis was performed to identify independent predictors of in-hospital pericardial effusion among young patients with acute myocardial infarction using variables that were significant in univariate analysis together with clinically relevant covariates.

In Model 1, lower BMI (OR: 0.92, 95% CI: 0.87–0.96, *P* < 0.001), male sex (OR: 0.50, 95% CI: 0.26–0.94, *P* = 0.032), Killip class ≥ II (OR: 3.14, 95% CI: 2.19–4.49, *P* < 0.001), STEMI (OR: 1.59, 95% CI: 1.08–2.35, *P* = 0.020), and higher hs-CRP levels (OR: 1.15, 95% CI: 1.09–1.21, *P* < 0.001) were independently associated with in-hospital pericardial effusion (Table 3).

**Table 3:** Multivariate logistic regression analysis of independent predictors for in-hospital pericardial effusion in young acute myocardial infarction patients.

Variables	Model 1		Model 2	
	OR (95% CI)	*P*	OR (95% CI)	*P*
BMI, kg/m²	0.92 (0.87, 0.96)	<0.001	1.42 (1.31, 1.53)	<0.001
Male, *n* (%)	0.50 (0.26, 0.94)	0.032	-	
Killip Class ≥ II	3.14 (2.19, 4.49)	<0.001	-	
STEMI	1.59 (1.08, 2.35)	0.020	0.63 (0.43, 0.93)	0.021
hs-CRP (mg/L)	1.15 (1.09, 1.21)	<0.001	-	
LVEF (per 1% decrease)	-		1.27 (1.06, 3.42)	0.023
PCT	-		1.87 (1.08, 3.23)	0.025
eGFR (per 10 units increase)	-		0.80 (0.67, 0.95)	0.012
TC	-		1.12 (1.03, 1.21)	0.007
lg (NT-proBNP/ALB ratio)	-		3.23 (1.30, 5.78)	<0.001

• Model 1: adjusted for BMI, sex (male), Killip class ≥ II, STEMI, and hs-CRP; Model 2: adjusted for BMI, STEMI, LVEF, PCT, eGFR, TC, and lg (NT-proBNP/ALB ratio).

• BMI, body mass index; STEMI, ST-segment elevation myocardial infarction; hs-CRP, high-sensitivity C-reactive protein; LVEF, left ventricular ejection fraction; PCT, plateletcrit; eGFR, estimated glomerular filtration rate; TC, total cholesterol; NT-proBNP, N-terminal pro-B-type natriuretic peptide; ALB, albumin; OR, odds ratio; CI, confidence interval.

In the extended multivariable model (Model 2), BMI (OR: 1.42, 95% CI: 1.31–1.53, *P* < 0.001), STEMI (OR: 0.63, 95% CI: 0.43–0.93, *P* = 0.021), lower LVEF (per 1% decrease; OR: 1.27, 95% CI: 1.06–3.42, *P* = 0.023), PCT (OR: 1.87, 95% CI: 1.08–3.23, *P* = 0.025), lower eGFR (per 10-unit increase; OR: 0.80, 95% CI: 0.67–0.95, *P* = 0.012), higher total cholesterol (OR: 1.12, 95% CI: 1.03–1.21, *P* = 0.007), and lg (NT-proBNP/ALB ratio) (OR: 3.23, 95% CI: 1.30–5.78, *P* < 0.001) were independently associated with in-hospital pericardial effusion (Table 3). Among these variables, lg (NT-proBNP/ALB ratio) showed a particularly strong association with the risk of in-hospital pericardial effusion.

**Figure 3:**
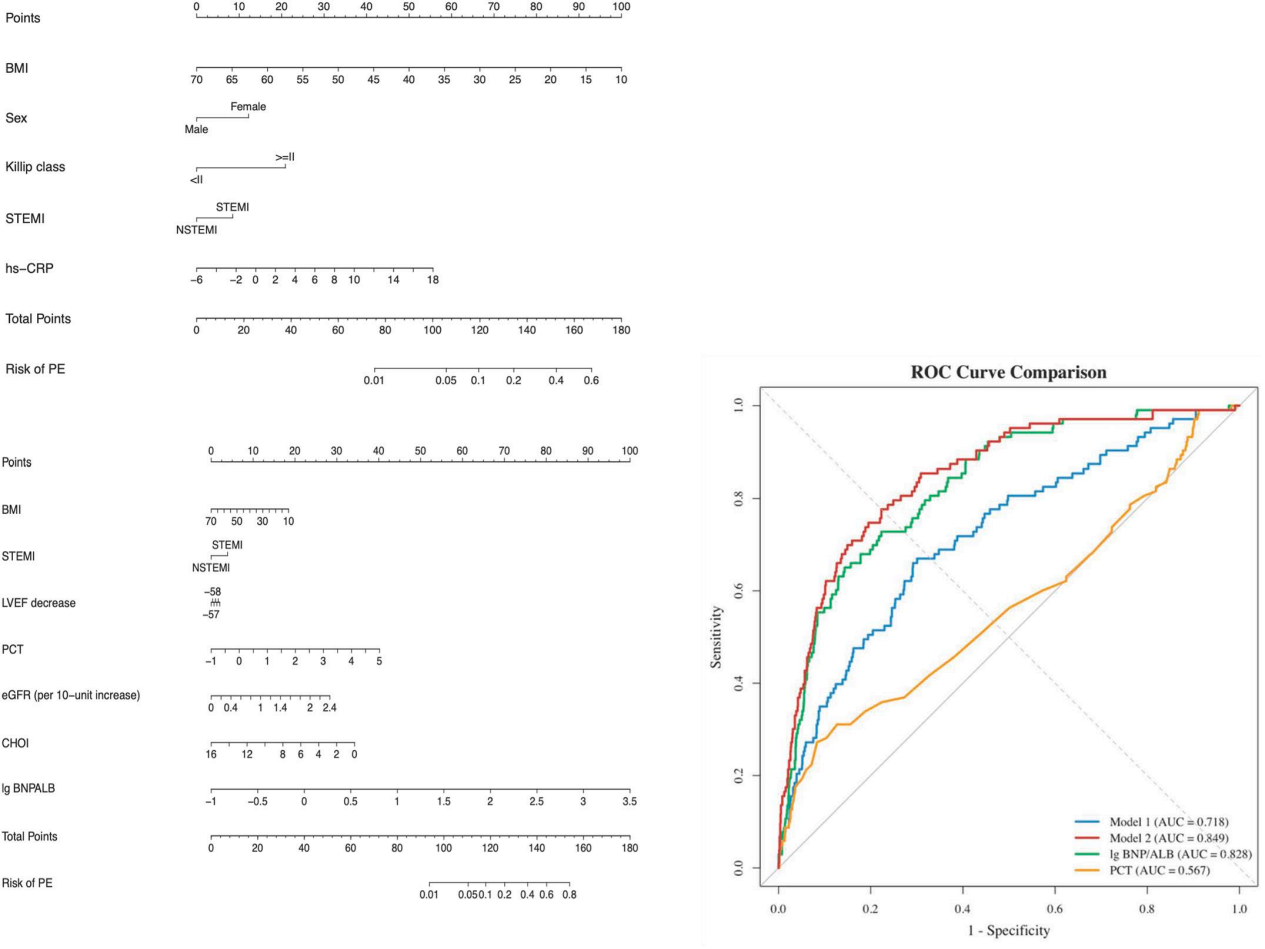
Comparison of the discriminative performance between the baseline and the full predictive models (A) Receiver Operating Characteristic (ROC) curves for the prediction of in-hospital pericardial effusion in young AMI patients. Blue Line (Model 1): The baseline clinical model including Age, Killip class, STEMI diagnosis, BMI, Sex, and hs-CRP, which yielded an AUC of 0.718 (sensitivity: 0.670, specificity: 0.699, Youden index: 0.369 at the optimal probability cut-off of 0.0728). Red Line (Model 2): The full integrated model derived by adding lg (NT-proBNP/Albumin ratio), Plateletcrit (PCT), eGFR, Total Cholesterol, and LVEF to the baseline model. The full model demonstrated a significantly higher AUC compared to the baseline model (0.849 vs. 0.718, *P* < 0.001 by DeLong test), indicating superior predictive accuracy. Furthermore, at its optimal cut-off of 0.0729, Model 2 achieved an improved sensitivity of 0.777, and a Youden index of 0.554. Abbreviations: AUC, Area Under the Curve; BMI, Body Mass Index; eGFR, estimated Glomerular Filtration Rate; LVEF, Left Ventricular Ejection Fraction; PCT, Plateletcrit.

To further evaluate the incremental predictive value of the novel biomarkers, we compared the baseline model (Model 1: BMI, sex, Killip class ≥ II, STEMI, and hs-CRP) with the extended model [Model 2: BMI, STEMI, LVEF, PCT, eGFR, CHOL, and lg (NT-proBNP/ALB ratio)]. As shown in Figure 3, the extended model demonstrated significantly better discriminative ability than the baseline model, with the AUC increasing from 0.718 to 0.849 (*P* < 0.001, DeLong test). Among the individual biomarkers, lg (NT-proBNP/ALB ratio) alone showed good predictive performance (AUC = 0.828), whereas PCT alone had limited discriminative ability (AUC = 0.567).

Furthermore, reclassification analysis showed that the addition of lg (NT-proBNP/ALB ratio) and PCT significantly improved risk stratification beyond the baseline model, with an NRI of 0.2198 (*P* < 0.001) and an IDI of 0.1116 (*P* < 0.001). These findings indicate that incorporation of the combined biomarkers meaningfully enhanced the ability to distinguish patients with and without in-hospital pericardial effusion. In sensitivity analysis, LASSO logistic regression with 10-fold cross-validation yielded comparable predictive performance (AUC = 0.842), and lg (NT-proBNP/ALB ratio) was retained as a key predictor, further supporting the robustness of the primary findings. (Figure 3)

## 4 Discussion

The present single-center retrospective study systematically evaluated the association between the admission NT-proBNP/ALB ratio (lg BNP/ALB) and in-hospital PE in young AMI patients aged <45 years, and further explored the incremental predictive value of this composite biomarker combined with PCT for PE. The main findings are as follows: (1) The incidence of in-hospital PE in this cohort was 7.1%, and PE patients had more severe clinical conditions, higher myocardial injury and inflammatory marker levels, and worse cardiac and renal function at admission; (2) lg BNP/ALB and PCT were independently associated with in-hospital PE and may assist in early risk stratification with lg BNP/ALB being the strongest predictor; Despite the relatively limited number of outcome events, the number of predictors included in the multivariable model was restricted to maintain model stability, and all selected variables were clinically relevant, supporting the robustness of the findings. (3) Incorporating lg BNP/ALB and PCT into the baseline clinical model significantly improved the discriminative ability and risk stratification accuracy for PE (AUC increased from 0.718 to 0.849, with significant NRI and IDI values).

The development of PE after AMI is a complex multifactorial pathological process involving cardiac hemodynamic changes, increased vascular permeability, and local/systemic inflammation (5). The present findings suggest that the lg BNP/ALB ratio, as a composite biomarker, can comprehensively reflect these interconnected pathological mechanisms, which is the fundamental reason for its superior predictive value over single biomarkers. On the one hand, NT-proBNP is a classic marker for ventricular wall stress and cardiac hemodynamics: AMI-induced myocardial necrosis increases left ventricular wall stress and intracardiac filling pressure, stimulating massive NT-proBNP secretion by cardiomyocytes. Elevated NT-proBNP levels are closely correlated with infarct size and hemodynamic disturbance, and the increased hydrostatic pressure in the coronary microvasculature further promotes fluid filtration from the vascular space to the pericardial cavity (9, 14, 15). On the other hand, serum ALB is a key negative acute-phase protein and the main determinant of plasma colloid osmotic pressure: severe systemic inflammation in the acute phase of AMI inhibits hepatic albumin synthesis, leading to hypoalbuminemia. This not only reflects inflammatory severity but also reduces plasma colloid osmotic pressure, which together with inflammation-induced increased vascular permeability accelerates fluid leakage into extravascular spaces including the pericardial cavity (10, 16). By integrating these two parameters, the NT-proBNP/ALB ratio simultaneously reflects hemodynamic load and inflammatory-nutritional imbalance in young AMI patients, thus more comprehensively and accurately predicting PE risk caused by the combined action of these two pathological mechanisms. The multivariate regression result (lg BNP/ALB adjusted OR: 3.23, *P* < 0.001) confirms this conclusion, which is consistent with research on the NT-proBNP/ALB ratio in other cardiovascular diseases such as chronic heart failure (11, 12, 17), further extending the clinical application of this composite biomarker to PE prediction in young AMI patients.

This study also identified PCT as an independent risk factor for in-hospital pericardial effusion in young patients with AMI (adjusted OR: 1.87, *P* = 0.025), representing a novel finding with important clinical implications. PCT, calculated as platelet count × mean platelet volume/10,000, reflects the total circulating platelet mass and provides a more comprehensive assessment of platelet activation status than either parameter alone. Compared with traditional platelet indices, PCT integrates both quantitative and morphological platelet characteristics, thereby better capturing platelet reactivity and thrombotic potential (18). Previous studies have demonstrated that elevated PCT is associated with increased platelet aggregation, heightened inflammatory activity, and adverse cardiovascular outcomes. In the context of acute myocardial infarction, particularly in younger patients where thrombus formation is often driven by plaque erosion and endothelial dysfunction, platelet activation plays a pivotal role in triggering local thrombo-inflammatory cascades. Therefore, PCT may serve as an integrated biomarker linking thrombosis and inflammation, two key mechanisms involved in the development of pericardial effusion (19, 20). The pathophysiology of AMI in young patients often differs fundamentally from that in older cohorts; it is more frequently driven by plaque erosion rather than traditional stable atherosclerotic plaque rupture (21). Plaque erosion is a highly platelet-dependent process where intact but dysfunctional endothelium triggers acute, localized thrombosis. Consequently, young patients—with their robust immune systems—are highly susceptible to an exaggerated “inflammatory storm” driven by the intense interaction between activated platelets and the innate immune system. In this context, platelets act as the primary nexus of “immuno-thrombosis.” Activated platelets release a variety of proinflammatory mediators (e.g., platelet factor 4, tumor necrosis factor-α, interleukin-6) and directly interact with circulating leukocytes to promote the formation of neutrophil extracellular traps (NETs) (22). This intense immuno-thrombotic cascade damages the vascular endothelial barrier, significantly increases microvascular permeability, and triggers massive local inflammatory exudation into the pericardial cavity. Compared to established systemic inflammatory markers such as the neutrophil-to-lymphocyte ratio (NLR), which primarily reflects downstream immune cell mobilization, PCT captures the upstream, initiating platelet-driven thrombotic and inflammatory mechanisms specific to young AMI patients. Therefore, an elevated PCT level more accurately mirrors this heightened state of immuno-thrombosis, providing a mechanistic link to the increased risk of in-hospital PE.

Consistent with previous studies (7, 23), Killip class ≥ II and decreased eGFR were also identified as independent PE risk factors. Killip class ≥ II indicates acute heart failure after AMI, which is closely associated with severe hemodynamic disturbance and increased intracardiac pressure, further promoting pericardial fluid exudation. Decreased eGFR reflects impaired renal function, which leads to fluid and sodium retention, increased blood volume and cardiac preload, thus elevating PE risk (4). Additionally, both univariable and multivariable analyses showed that higher total cholesterol levels were associated with an increased risk of in-hospital pericardial effusion. Although the underlying mechanism remains unclear, this finding may reflect the complex metabolic and inflammatory alterations accompanying acute myocardial infarction. Given the observational nature of the study, the association between total cholesterol and pericardial effusion should be interpreted with caution and warrants further investigation. Interestingly, BMI showed a direction change between the unadjusted and adjusted models. While higher BMI appeared protective in the initial model, it was associated with increased risk after full adjustment. This finding may reflect the influence of confounding factors such as disease severity and nutritional status, commonly described as the “obesity paradox” in cardiovascular diseases. After accounting for key variables including cardiac function and systemic inflammation, BMI may better represent its underlying biological effect rather than being a surrogate of clinical severity. A similar directional change was observed for STEMI. While STEMI was positively associated with in-hospital pericardial effusion in the univariable analysis and in Model 1, its association became inverse after full adjustment in Model 2. This shift may reflect the influence of collinearity and overlap with other markers of infarct severity and cardiac dysfunction, particularly LVEF and eGFR, which were incorporated into the extended model. Accordingly, this finding should be interpreted cautiously and warrants further validation in external cohorts.

Young AMI patients generally have a better short-term survival rate than the elderly, but in-hospital PE can significantly complicate the clinical course and increase the risk of adverse events such as cardiac tamponade and sudden cardiac death. Thus, early identification of high-risk patients is key to improving the clinical prognosis of young AMI patients. The NT-proBNP/ALB ratio and PCT are both derived from routine admission laboratory tests, with the advantages of easy detection, low cost, and high repeatability, making them highly suitable for routine clinical application. The study results show that incorporating these two biomarkers into the baseline clinical model significantly improves PE predictive accuracy (AUC from 0.718 to 0.849), and reclassification analysis confirms the significant improvement in risk stratification ability. To translate these findings into clinical practice, these findings may support closer echocardiographic surveillance and more individualized monitoring strategies in high-risk young AMI patients. Upon admission, clinicians can leverage the optimal cut-off value of the lg BNP/ALB ratio, in conjunction with procalcitonin (PCT) levels, to stratify young AMI patients into high-risk and low-risk cohorts with high accuracy. For low-risk individuals, adherence to guideline-recommended standard post-AMI care—encompassing antiplatelet therapy, beta-blockers, statins, and routine clinical monitoring—remains sufficient to ensure favorable outcomes. In contrast, high-risk patients necessitate a rigorous, protocolized intensive monitoring regimen, including targeted echocardiographic assessments performed daily or every other day during the highly vulnerable acute phase (i.e., the first 3–7 days post-infarction). This frequent imaging surveillance is critical for the early detection of subclinical fluid accumulation, a harbinger of impending hemodynamic compromise.

Furthermore, this validated predictive model provides clear guidance for defining therapeutic intervention thresholds. Identification of a patient as high-risk may prompt clinicians to consider preemptive anti-inflammatory therapies—such as colchicine or select non-steroidal anti-inflammatory drugs (NSAIDs), when not strictly contraindicated by comorbidities (e.g., severe hepatic and renal insufficiency, active bleeding)—to mitigate the impending systemic inflammatory storm associated with adverse post-AMI outcomes. Additionally, it serves as an early alert to the structural heart team regarding the potential for rapid hemodynamic deterioration, thereby lowering the clinical threshold for timely therapeutic pericardiocentesis if subtle signs of impending cardiac tamponade emerge (e.g., worsening dyspnea, hypotension, elevated jugular venous pressure).

Ultimately, the integration of this composite biomarker-based stratification strategy embodies the principles of precision medicine in acute cardiovascular care. By enabling targeted resource allocation—directing intensive monitoring and preemptive interventions to those at highest risk while avoiding unnecessary overtreatment in low-risk patients—it optimizes the efficiency of clinical management and effectively reduces the incidence of life-threatening pericardial effusion (PE) and related complications in young AMI patients.

## 5 Limitations

This study has several limitations. First, as a single-center retrospective investigation, complete elimination of selection bias was not possible, and our findings require further validation in larger multicenter cohorts. Second, we lacked direct measurements of infarct size. Although we adjusted for established surrogate markers, including LVEF, Killip class, STEMI status, and inflammatory markers, to account for infarct severity, residual confounding may still exist. In particular, LVEF and Killip class, while widely used surrogate indicators, capture different dimensions of infarct severity compared with peak troponin levels or cardiac MRI, and therefore may not fully account for the variance explained by direct infarct size measurements. Third, because the timing and frequency of repeated echocardiographic examinations were not standardized in this retrospective study, delayed or mild pericardial effusions may have been under detected, which could introduce misclassification bias. Fourth, the biochemical characterization of pericardial fluid (e.g., hemorrhagic vs. serous) was not available, as invasive procedures such as pericardiocentesis are not routinely performed for mild-to-moderate effusions. Finally, our analysis was restricted to in-hospital outcomes; the long-term prognostic implications of pericardial effusion in this young population remain to be fully elucidated.

## 6 Conclusion

The NT-proBNP/ALB ratio was independently associated with in-hospital pericardial effusion in young AMI patients after adjustment for cardiac function and infarction severity. Incorporation of NT-proBNP/ALB and PCT improved model discrimination and risk stratification. As a readily available biomarker, NT-proBNP/ALB may aid early identification of high-risk patients. Prospective multicenter validation is warranted.

## Data Availability

The original contributions presented in the study are included in the article/supplementary material, further inquiries can be directed to the corresponding author.
